# Increased Tacrolimus Exposure in Kidney Transplant Recipients With COVID-19: Inflammation-Driven Downregulation of Metabolism as a Potential Mechanism

**DOI:** 10.3389/ti.2022.10269

**Published:** 2022-05-16

**Authors:** Sylvia D. Klomp, Soufian Meziyerh, Maurits F. J. M. Vissers, Dirk J. A. R. Moes, Eline J. Arends, Y. K. Onno Teng, Jesse J. Swen, Aiko P. J. de Vries

**Affiliations:** ^1^ Department of Clinical Pharmacy and Toxicology, Leiden University Medical Center, Leiden, Netherlands; ^2^ Leiden Network for Personalised Therapeutics, Leiden University Medical Center, Leiden, Netherlands; ^3^ Department of Internal Medicine, Division of Nephrology, Leiden University Medical Center, Leiden, Netherlands; ^4^ Leiden Transplant Center, Leiden University Medical Center, Leiden, Netherlands; ^5^ Centre for Human Drug Research, Leiden, Netherlands; ^6^ Leiden University Medical Center, Leiden, Netherlands

**Keywords:** COVID-19, kidney transplant, Tacrolimus, metabolism, CYP3A, phenoconversion

## Abstract

Kidney transplant recipients (KTRs) are at increased risk of severe COVID-19 disease compared to the general population. This is partly driven by their use of immunosuppressive therapy, which influences inflammatory responses and viral loads. Current guidelines suggest to withdraw mycophenolate while calcineurin inhibitors are often continued during a COVID-19 infection. However, clinical signs of calcineurin toxicity have been described in multiple COVID-19 positive KTRs. In this report we describe the course of tacrolimus exposure prior to, during, and post COVID-19 in observations from three clinical cases as well as four KTRs from a controlled trial population. We postulate inflammation driven downregulation of the CYP3A metabolism as a potential mechanism for higher tacrolimus exposure. To mitigate the risk of tacrolimus overexposure and toxicity therapeutic drug monitoring is recommended in KTRs with COVID-19 both in the in-, out-patient and home monitoring setting.

## Graphical Abstract

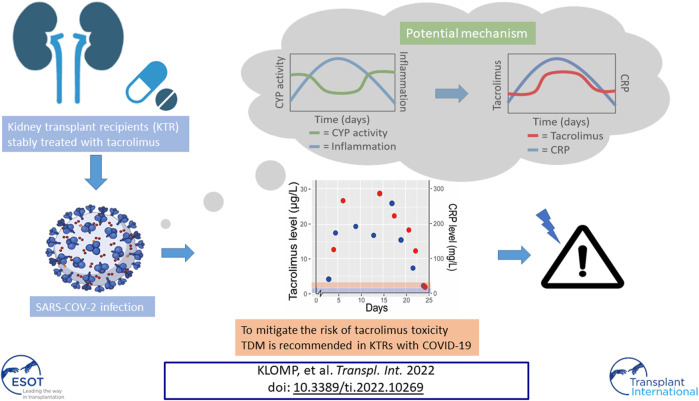

## Introduction

Kidney transplant recipients (KTRs) with severe acute respiratory syndrome due to coronavirus-2 (SARS-COV-2) are at threefold increased risk of a severe course of coronavirus disease‐2019 (COVID-19) (1). Immunosuppressive therapy to prevent transplant rejection may diminish anti-viral immunity potentially causing higher viral loads and a longer time-to-negativity for SARS-CoV-2 nucleic acid testing in nasopharyngeal swabs although it may also protect against an overshooting immune response (2, 3). Most KTRs are on a triple immunosuppressive maintenance regime consisting of the antimetabolite mycophenolate mofetil (MMF), a calcineurin inhibitor (CNI) and prednisolone (4). In SARS-CoV-2 positive KTRs, consensus guidelines recommend to withdraw MMF in low immunological risk patients, partly based on experience with the influenza H1N1 pandemic (5, 6). By contrast, CNIs are usually continued as these can be more closely titrated via therapeutic drug monitoring (TDM). Moreover, CNIs may have *in-vitro* activity against SARS-CoV2 (7). During the COVID-19 pandemic in Europe thousands of KTRs have been diagnosed with COVID-19 (8). At the Leiden University Medical Center all KTRs with mild COVID-19 symptoms were home-monitored with a “COVID-box” which included a blood pressure monitor, a thermometer, and a pulse oximeter to combine subjective and objective parameters for adequate monitoring (9).

Observations from our clinical practice raised the suspicion that KTRs, who contracted COVID-19, developed signs of tacrolimus toxicity including complaints of tremors, hypertension, and headaches. This has also been described in other non-controlled cohorts (10, 11)**.**

With this study, we aimed to describe dynamics in tacrolimus exposure in KTRs that contracted COVID-19. Data on inflammatory response and tacrolimus exposure prior to, during, and after COVID-19 have been analysed for three clinical cases. Subsequently we decided to analyse these changes in tacrolimus exposure in a controlled trial population with less severe disease to confirm both our findings and those in other beforementioned cohorts (10, 11).

## Methods

### Patient Selection and Data Collection

During the different waves of the pandemic, our general clinical impression was that a majority of our KTRs had increased tacrolimus levels with clinical symptoms of toxicity. For a first exploration on the relationship between tacrolimus exposure and COVID-19 infection, we describe three clinical cases that developed a toxic tacrolimus trough concentration (C_trough_) above 20 μg/L (target range 5–7 μg/L > 6 weeks posttransplant) at the time of hospitalisation or hospital visit for respiratory insufficiency caused by COVID-19. To explore a possible correlation with inflammation status, C-reactive protein (CRP) was used as biomarker. Case selection was based on the availability of patient consent, availability of tacrolimus C_trough_ and CRP levels before, during and after COVID-19 infection, and lack of a clear explanation for the tacrolimus concentration increase including drug-drug interactions (DDIs) and/or recent dose adjustments. Tacrolimus C_trough_ obtained during COVID-19 infection were compared to recent pre-COVID tacrolimus C_trough_ of the cases in combination with CRP concentrations.

As this clinical case selection inevitably leads to selection bias (e.g., towards most critically ill KTRs), we also investigated data from a randomized clinical trial (RCT) population (VOCOVID trial, NCT04701528) to assess the impact of COVID-19 on tacrolimus exposure. In this prospective open label trial, KTRs on maintenance immunosuppression with tacrolimus (+ mycophenolic acid and/or prednisolone and/or everolimus) with COVID-19 (confirmed by NAT) were randomized between continuation of tacrolimus and prednisolone (as standard of care during a COVID-19 infection) or replacement of tacrolimus by voclosporine (because of possible favourable anti-viral properties). Patients randomized to voclosporine are outside the scope of this article due to absence of voclosporine measurements prior to COVID-19.

Within this RCT, KTRs were followed up on a regular basis with C_trough_ measurements on day 4, 8, and 28, and AUC measurements on day 8 and day 28 using a dried-blood-spot TDM technique (12) which provides extensive insight in pharmacokinetics during and post COVID-19. During these visits multiple laboratory measurements have been performed including CRP concentrations.

For all cases demographic, clinical, pharmacologic (tacrolimus daily dose and immunosuppressive regimen), and laboratory measurements (CRP, CYP3A5 genotype, tacrolimus C_trough_ and AUC, and NAT) data were retrospectively collected from electronic health records.

### Definitions and Statistical Analysis

For the clinical cases, post-COVID-19 status was defined as the period following the first negative nucleic acid test (NAT) after a confirmed COVID-19 infection in combination with resolution of symptoms. For the RCT cases, post COVID-19 was defined as resolution of symptoms in combination with high cycle threshold-values (>34) in NAT for nasopharyngeal swabs. CRP concentrations, tacrolimus C_trough_, and AUCs obtained during COVID-19 infection were dose corrected and compared to pre-COVID-19 values. Descriptive statistics and graphical representation were used to summarize each patient’s course. Categorical data was presented using frequencies and percentages, while continuous data was presented as means and ranges. Due to the small sample size of seven patients, no formal statistical analyses were performed.

## Results

### Cases From Clinical Practice

**FIGURE 1:**
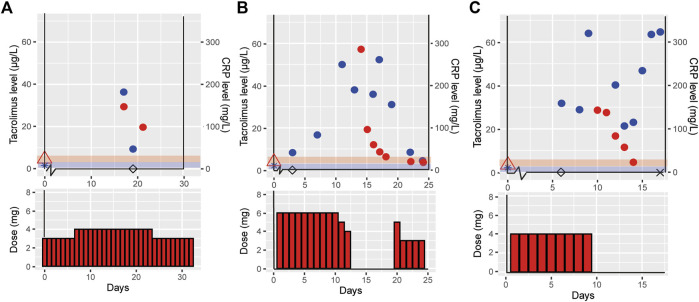
Effect of COVID-19 on tacrolimus pharmacokinetics in three clinical cases. This figure shows the tacrolimus, C-reactive protein (CRP) levels and the tacrolimus dose for the three different cases case 1 (outpatient) **(A)**, case 2 (hospital admission with recovery) **(B)** and case 3 (hospital admission resulting in death) **(C)**. Day 0 is defined as the start of COVID-19 symptoms, tacrolimus (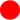), CRP (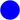), tacrolimus level prior to COVID-19 (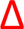), CRP level prior to COVID-19 (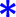), day of positive. SARS-CoV-2 PCR test (◊), day the patient died (×), the red and blue shades indicate the therapeutic range of tacrolimus and normal CRP levels, respectively.

For the three selected clinical cases, disease status, CYP3A5 genotype, tacrolimus treatment dose and exposure prior to infection, during and post COVID-19 infection are shown in Table 1. Figure 1 depicts tacrolimus C_trough_ concentrations prior to and during COVID-19 infection and available CRP concentrations.

**TABLE 1:** Demographics and tacrolimus dose and exposure prior to, during and post COVID-19 infection of three clinical cases.

Case	Age (y), sex	Status during COVID-19 & disease Severity^	CYP3A5 Genotype	Clinical Symptoms of TAC Toxicity	Immunosuppressive therapy prior to COVID-19 Onset	Day of Positive NAT-test (first Symptoms Started at D0)	Parameter	Prior to COVID-19	During COVID-19	Post COVID-19
1	57, male	Outpatient, Severe disease	*3/*3	Tremors and acute kidney injury (30% increase in baseline creatinine)	Tac bid 1.5 mg	20	Disease day		17	21
					Pred qd 5 mg		CRP (mg/L)		181.7	46.8
					MPA bid 500 mg (day 1–6)		Tac day dose (mg)	3	4	4
							C_trough_ (µg/L)	5.8	29.4	19.7
							C_trough_/dose(µg/L*mg)	1.9	7.4	4.9
2	67, male	Hospital admission, Severe disease	*3/*3	Headache, tremors and acute kidney injury (45% increase in baseline creatinine)	Tac bid 3 mg	3	Disease day		14	24
					Pred qd 5 mg		CRP (mg/L)		189.9	22.2
							Tac day dose (mg)	6	0	3
							C_trough_ (µg/L)	7.2	57.2	3.6
							C_trough_/dose(µg/L*mg)	1.2	∞	1.2
3	56, female	Hospital admission followed by death due to respiratory insufficiency, Severe disease	*3/*3	Tremors and acute kidney injury (60% increase in baseline creatinine)	Tac qd 4 mg	6	Disease day		10	14
					Pred qd 5 mg		CRP (mg/L)		515	115
							Tac day dose (mg)	4	4	0
							C_trough_ (µg/L)	3.9	28.6	4.6
							C_trough_/dose(µg/L*mg)	0.98	7.15	∞

CRP, C-reactive protein; NAT, nucleic acid-test; NA, not applicable; MPA, methylphenolic acid; Tac, tacrolimus; Pred, prednisolone.

Disease day starts at the day of symptom onset. C_trough_/Dose = the dose-corrected C_trough_ calculated as C_trough_ divided by the total daily dose. Prior to COVID-19, is defined as the most recent available exposure in the previous year. After COVID-19 is defined as an exposure after a negative NAT. The total daily dose is calculated by adding the morning and evening dose in case of BID dosing.

∞indicates dividing by zero, due to tacrolimus discontinuation.

^according to WHO, classification.

Case 1, a 57 years old male, reported COVID-19 symptoms including fever, cough, dyspnea, tremors and an acute kidney injury with 30% increase from baseline creatinine. There were no signs of liver dysfunction (AST 36 U/L and ALT 28 U/L). He visited the clinic two times during his disease course, but was not admitted to the hospital. Pre-COVID-19 he was treated with 3 mg tacrolimus (1.5 mg BID) and during COVID-19 the daily dose was 4 mg tacrolimus. He received no medication that is known to cause any DDI with tacrolimus. During COVID-19 a toxic tacrolimus C_trough_ level of 29.4 μg/L was measured, resulting in a 289% increased tacrolimus dose-corrected C_trough_ (C_trough_/Dose) during COVID-19 infection as compared to pre-COVID-19 (tacrolimus C_trough_ of 5.8 μg/L) (Table 1 and Figure 1A). Post COVID-19, the patient recovered well, all laboratory measurements returned back to within normal ranges and the COVID-19 symptoms disappeared.

Case 2, a 67 years old male, presented with COVID-19 symptoms of fever, headache, dyspnea, tiredness, and chest pains. At COVID-19 disease day 11 he was admitted to the hospital, because of respiratory insufficiency requiring additional oxygen suppletion, and with a CRP level of 249.9 mg/L. His liver enzymes were elevated, AST and ALT rose to 117 and 122 U/L, respectively. He was on maintenance immunosuppression with 3 mg tacrolimus BID (6 mg/day). Therapy with hydrochloroquine was initiated on disease day 11 and stopped at day 16. Two weeks after disease onset, he had a tacrolimus C_trough_ of 57.2 μg/L and a CRP level of 189.9 mg/L (Table 1 and Figure 1B). Signs of tacrolimus intoxication included a headache and tremor. Tacrolimus was temporarily interrupted for 6 days, during which C_trough_ declined to 6.3 μg/L. Tacrolimus was reinitiated at a conservative dose of 1.5 mg BID, half of the original dose. At disease day 25 he was discharged.

Case 3, a 56 years old female, experienced COVID-19 symptoms including, fever, nausea, cough and dyspnea. At day 8 post-positive COVID-19 PCR she was admitted to the intensive care unit (ICU) due to respiratory insufficiency requiring mechanical ventilation. She had elevated liver enzymes, AST and ALT levels of 105 and 76 U/L, respectively. She was on maintenance immunosuppression with 4 mg tacrolimus qd. Hydroxychloroquine was initiated on disease day 6 an stopped at day 8. At day 10, a tacrolimus C_trough_ of 28.6 μg/L was measured with a CRP level of 515 mg/L, a 630% higher dose-corrected C_trough_ during COVID-19 compared to pre-COVID-19 (Table 1 and Figure 1C). At that point, tacrolimus was withdrawn due to tacrolimus intoxication diagnosis at ICU. The patient died from COVID-19 related complications on disease day 17.

### Case Series From the RCT VOCOVID

From November 2020 until February 2021 eight KTRs were included in the VOCOVID trial, of which five were randomized to continue tacrolimus and three switched to voclosporine. One of the five tacrolimus continuers was excluded from our analysis due to lack of available AUC measurements, since the patient died early from COVID-19, leaving a total of 4 cases. The association between tacrolimus C_trough_ and CRP of this fifth case is, nonetheless, depicted in Supplementary Figure S1.

**FIGURE 2:**
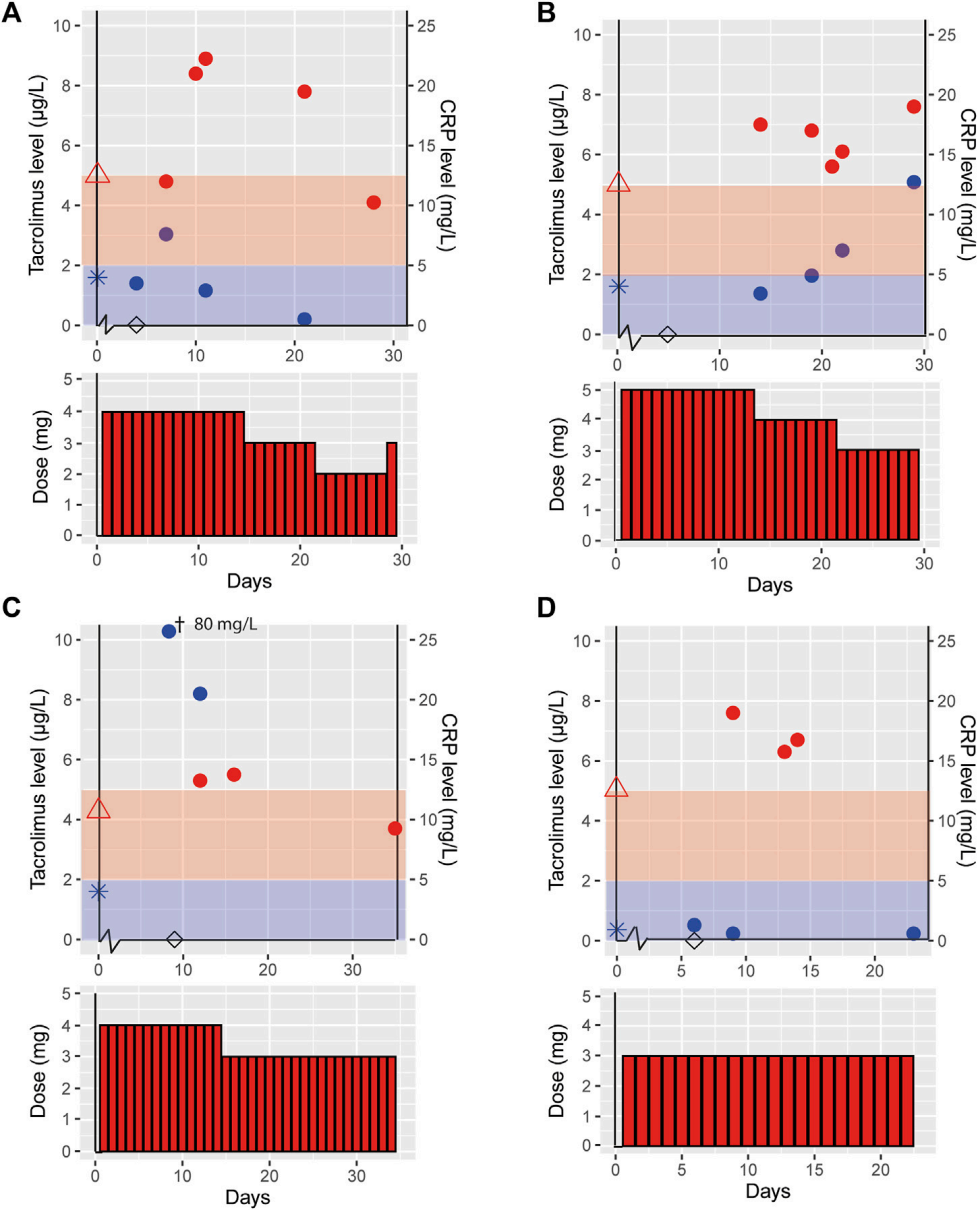
Effect of COVID-19 on tacrolimus pharmacokinetics in patients participating in a clinical trial. This figure shows the tacrolimus, C-reactive protein (CRP) levels and the tacrolimus dose for the cases participating in the control arm of the VOCOVID trial, case A **(A)**, B **(B)**, C **(C)** and D **(D)**. Day 0 is defined as the start of COVID-19 symptoms, tacrolimus (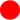), CRP (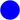), tacrolimus level prior to COVID-19 (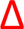), CRP level prior to COVID-19 (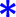), day of positive SARS-CoV-2 PCR test (◊), the red and blue shades indicate the therapeutic range of tacrolimus and normal CRP levels, respectively. ^†^This measurement is out of axis reach, the value is 80 mg/L.

Figure 2 illustrates tacrolimus exposure for VOCOVID trial cases (Case A, B, C and D) both prior to and during COVID-19, and available CRP concentrations.

These four cases had an age range of 47–57 years and two were male. Case C was a heterozygous CYP3A5 expressor (CYP3A5*1/*3, resulting in increased tacrolimus metabolism thus requiring a higher daily dose at baseline), case A a non-expressor (CYP3A5*3/*3) and cases B and D were not genotyped (Table 2). All trial cases were more than 3 years post transplantation and on a stable tacrolimus dose prior to contracting COVID-19. Tacrolimus dose and exposure prior to infection, during and post COVID-19 infection are shown in Table 2. Case A reported minor and temporary short-lasting minor, self-limiting gastro-intestinal complaints with two loose stools per day without vomiting. Other symptoms were all related to the SARS-CoV-2 infection. All trial cases had complaints of fever, dyspnea and coughing. None of the trial cases required hospitalization for additional oxygen suppletion. Case B had slightly increased liver enzymes; AST was 38 U/L and ALT 73 U/L. All other trial cases had liver enzymes laboratory values with the normal reference range.

**TABLE 2:** Demographics and tacrolimus dose and exposure prior to, during and post COVID-19 infection of four cases within the VOCOVID trial.

Case	Age (y), sex	Status during COVID-19 & disease Severity^	CYP3A5 Genotype	Clinical Symptoms of TAC Toxicity	Immunosuppressivetherapy prior to COVID-19 Onset	Day of Positive NAT-test (first Symptoms Started at D0)	Parameter	Prior to COVID-19	During COVID-19^$^	Post COVID-19
A	47, male	Outpatient, Mild disease	*3/*3	Hypertension, tremors and acute kidney injury (20% increase in baseline creatinine)	Tac qd 4 mg	4	Disease day		10	30
					Pred qd 5 mg		CRP (mg/L)		7.6	1.0
							Tac day dose (mg)	4	4	3
							AUC (µg*h/L) #	180	236	114
							AUC/dose(µg*h/L*mg)	45	59	38
B	47, female	Outpatient, Mild disease	NA	Hypertension and tremors	Tac qd 5 mg	5	Disease day		21	42
					Pred qd 5 mg		CRP (mg/L)		7.0	3.8
					Evl bid 4 mg (discontinued on D13)		Tac day dose (mg)	5	4	2.5
							AUC (µg*h/L)	149	164	103
							AUC/dose(µg*h/L*mg)	30	41	41
C	49, female	Outpatient, Moderate disease	*1/*3	None	Tac bid (2–3) mg	9	Disease day		16	35
					Pred qd 5 mg		CRP (mg/L)		20.5	NA
							Tac day dose (mg)	5	3	3
							AUC (µg*h/L)	80*	94	64
							AUC/dose(µg*h/L*mg)	16	31	21
D	57, male	Outpatient, Mild disease	NA	None	Tac bid 1.5 mg	2	Disease day		13	34
					Pred qd 5 mg		CRP (mg/L)		0.6	0.8
							Tac day dose (mg)	3	3	3
							AUC (µg*h/L)	77	111	69
							AUC/dose(µg*h/L*mg)	26	37	23

CRP, C-reactive protein; NAT, nucleic acid-test; NA, not applicable; AUC, area under the curve; Tac, tacrolimus; Pred, prednisolone; Evl, everolimus.

Disease day starts at the day of symptom onset. AUC/Dose = the dose-corrected AUC calculated as AUC, divided by the total daily dose. Prior to COVID-19, is defined as the most recent available exposure in the previous year. After COVID-19 is defined as an exposure after a negative NAT. The total daily dose is calculated by adding the morning and evening dose in case of BID dosing.

*This historic value was from 2012.

^$^This measurement is after the positive NAT.

^According to WHO, classification.

^#^The AUC, is calculated form Ctrough levels, C0, C2 and C3 and for dried-blood-spot from C0, C1, C2 and C3.

The VOCOVID cases displayed on average a 51% (range 31%–94%) higher tacrolimus dose-corrected AUC (AUC/Dose) during COVID-19 as compared to pre-COVID-19. Post-COVID-19 there was a 26% (range 0%–38%) decline in AUC/Dose compared to the situation during disease. At the latest available time point, approximately 1 month after COVID-19, AUC/Dose for cases A, C and D showed a return to baseline, suggesting a correlation between the increased exposure and COVID-19. AUC/Dose at the latest available time point for case B was similar compared to AUC/Dose during infection. For this case, the AUC measurement during COVID-19 was obtained later in the disease course compared to the other trial cases (day 21 vs. day 10–16).

## Discussion

In this report, we could confirm observations of tacrolimus toxicity in both clinically admitted KTRs and outpatient managed KTRs who underwent protocolized TDM as part of a clinical trial. COVID-19 infection was associated in with higher tacrolimus levels (range 4%–794%) in our cases, which has previously only been described in hospitalized (and thus more ill) KTRs (10, 11). These data underpin the need of frequent TDM in all COVID-19 KTRs to prevent tacrolimus overexposure, regardless of time after transplantation and treatment status, which has important clinical ramifications, specifically for patients with mild COVID-19 related symptoms that do not require hospitalization. Tacrolimus is notorious for its highly variable PK and narrow therapeutic window in which toxic levels can result in result in life-threatening complications including renal failure, hypertension/thrombotic micro-angiopathy and/or neurotoxicity. Via AUC measurements we managed to get more insight in the potential cause of overexposure in KTRs with COVID-19.

The observed increase in tacrolimus concentrations during COVID-19 infection could potentially result from changes in absorption, metabolism, and excretion, possibly via DDIs.

First of all, tacrolimus absorption shows substantial inter- and intra-patient variability. Intra-patient variability can result from diarrhea, possibly explained by changed drug solubility and intestinal permeability (13, 14). In our study one case reported minor and temporary gastro-intestinal complaints with two loose stools per day, not expected to substantially change absorption (15), albeit we cannot exclude an effect from SARS-COV-2 on luminal cells (16). Notably, KTRs on cyclosporin also showed higher cyclosporin trough levels during COVID-19 infection, despite its characteristic of reduced absorption in diarrhea (13). Unfortunately, available cyclosporin data were too sparse to allow further analysis. In addition, a nil per os (nothing by mouth) status could affect tacrolimus absorption. With the exception of ICU case 3 who was intubated at the ICU, all cases were able to eat and drink by mouth. At the time of intubation, case 3 was no longer receiving tacrolimus.

Another potential cause of increased tacrolimus exposure is altered hepatic function or mucosal drug metabolism (13, 14) since tacrolimus is metabolized by CYP3A4 and CYP3A5 (17). All cases described within this case series did not have significant elevation of liver enzymes or bilirubin rendering hepatic dysfunction less probable. Furthermore, all RCT-cases were from the outpatient setting and remained outpatient during the whole study period, rendering (multi-) organ failure or significant liver dysfunction as possible explanation for tacrolimus toxicity unlikely.

Furthermore, there was no use of concomitant CYP3A4 inhibitors in our cases, which could also have explained higher tacrolimus exposure resulting from a DDI. Two of the clinical cases were shortly treated with hydroxychloroquine which, to our knowledge, does not inhibit CYP3A4 (unlike chloroquine). The initiation of hydroxychloroquine theoretically could have led to a slight elevation in tacrolimus C_trough_, (18) however the actual elevation observed (>700%) was much higher than what would; be expected based on literature (18) (Figure 1B). Besides, the other five cases did not use hydroxychloroquine and nonetheless showed increased tacrolimus exposure; which suggests that any influence from DDIs is either small or absent in our population.

It is also unlikely that the observed increase in tacrolimus concentrations are resulting from intra-patient variability. The variability of tacrolimus clearance in our center is known to be <20% [unpublished data] in accordance with the literature where the intra-patient variability is reported to be 17% between measurements for patients 6–12 months after renal transplantation (19, 20). The dose corrected tacrolimus concentrations show little variability during the COVID-19 episode in the clinical and trial cases (range 0.20–0.73 μg/L/mg Figures 1, 2), indicating low day to day intra-patient variability. Moreover, all cases showed a similar pattern of tacrolimus levels prior to, during and post COVID-19 indicating an effect of disease course (Figures 1, 2).

Contrarily, two of the clinical cases (case 2 and 3) temporarily interrupted tacrolimus treatment and showed a slow clearance [for example, the observed half-life for case 3 was >48 h, where normally this would be in the range of 12–15 h for tacrolimus (21)] (Figure 1). The slow decrease in tacrolimus concentration after cessation of tacrolimus points towards an impaired metabolism, since excretion of unmetabolized tacrolimus via feces only contributes to <1% of tacrolimus clearance (21). In addition, liver function enzymes ALT and AST did not indicate clinically relevant liver dysfunction further supporting our hypothesis of an alteration in CYP3A activity.

We observed that increase in dose corrected tacrolimus levels align with increase in CRP levels, especially in the cases admitted to hospital (Figures 1, 2). It has been shown that upregulation of interleukin (IL)-6 results in increased CRP levels in patients with acute inflammation (22), and CYP3A activity can be down-regulated by pro-inflammatory cytokines (22, 23), including tumor necrosis factor (TNF)-α, IL-1β, IL-6, IL-2, and interferon (IFN)-γ. Since COVID-19 has been found to elevate these pro-inflammatory cytokines this could lead to so called inflammatory based phenoconversion (24–26). Indeed, COVID-19 induced phenoconversion has previously been reported for a COVID-19 patient with clozapine toxicity (27). It was reported that inflammation potentially induced downregulation of CYP1A2. Similarly, increased tacrolimus levels can be the result of CYP3A downregulation via inflammatory based phenoconversion (28–30).

Of note, CRP is an imperfect marker for IL-6 or other inflammatory mediators. IL-6 levels were however unavailable and are not part of routine patient care. This would explain why increased tacrolimus exposure is not observed in transplant patients with comparable CRP levels for, e.g., septicaemia, where IL-6 or other inflammatory mediators are not expected to be elevated (21).

We, therefore, postulate that inflammation resulting from COVID-19 infection results in CYP3A phenoconversion in KTRs. COVID-19 may thus lead to unwanted higher exposure of tacrolimus, most likely caused by downregulated CYP3A metabolism by pro-inflammatory cytokines.

Our hypotheses may also account for the low rates of rejections during COVID-19 observed in our patients, despite reduction of immunosuppressive treatments. However, this needs to be investigated further in studies specifically designed for this purpose.

If our hypotheses of inflammation driven phenoconversion holds true, this may also be relevant for IL-6-inhibitors including tocilizumab and clazakizumab that are currently being introduced into the KTR population both in COVID-19 and chronic antibody-mediated rejection. Moreover, phenoconversion may also play a role during other infections associated with upregulation of proinflammatory cytokines, but this needs to be studied more before conclusions can be drawn.

In conclusion, tacrolimus exposure should be carefully monitored during COVID-19 to potentially prevent tacrolimus toxicity and a negative impact on cellular immunity and viral load in patients. A plausible cause for toxicity seems inflammation induced phenoconversion of CYP3A activity which needs to be confirmed in future studies.

## Data Availability

The original contributions presented in the study are included in the article/Supplementary Material, further inquiries can be directed to the corresponding authors.

## Supplementary Material

Supplementary Figure S1 Effect of COVID-19 on tacrolimus pharmacokinetics. This figure shows the tacrolimus, C-reactive protein (CRP) levels and the tacrolimus dose for the tacrolimus trial patient, admitted to the ICU. Day 0 is defined as the start of COVID-19 symptoms, tacrolimus () C-reactive protein (CRP) (), tacrolimus level prior to COVID-19 (), CRP level prior to COVID-19 (), day of positive SARS-CoV-2 PCR test (◊), day the patient died (×), the red and blue shades indicate the therapeutic range of tacrolimus and normal CRP levels, respectively.
