# Supplementary figures and images for: Increased Tacrolimus Exposure in Kidney Transplant Recipients With COVID-19: Inflammation-Driven Downregulation of Metabolism as a Potential Mechanism
Source: Transpl Int. 2022 May 16;35:10269. doi: 10.3389/ti.2022.10269 (PMC9148963; doi:10.3389/ti.2022.10269)

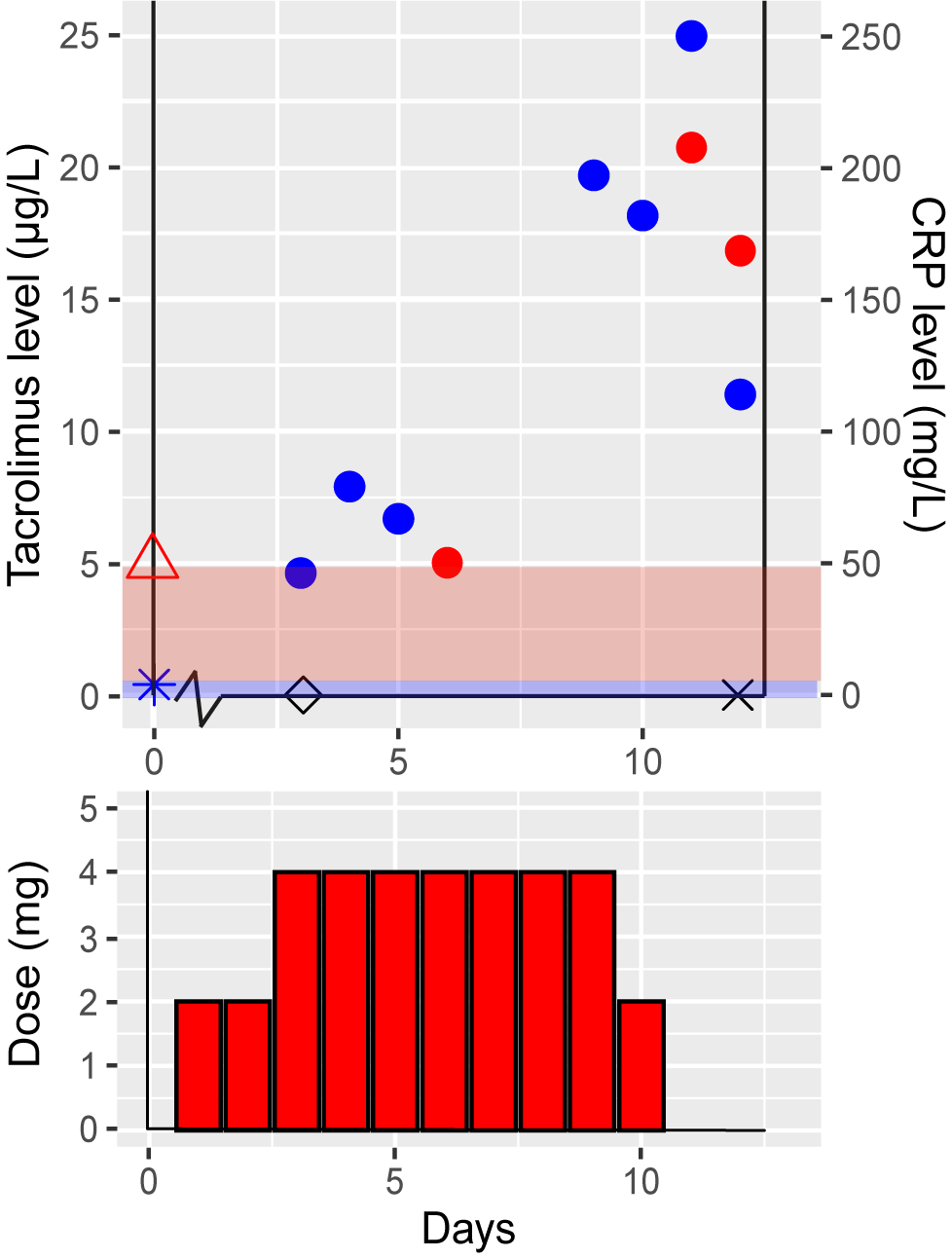

Supplement: Supplementary file 1 [file Image1.TIF]
